# Combined use of *N*-acetylcysteine and Liberase improves the viability and metabolic function of human hepatocytes isolated from human liver

**DOI:** 10.1016/j.jcyt.2014.01.006

**Published:** 2014-06

**Authors:** David C. Bartlett, James Hodson, Ricky H. Bhogal, Janine Youster, Phil N. Newsome

**Affiliations:** National Institute for Health Research Biomedical Unit and Centre for Liver Research, University of Birmingham, Birmingham, United Kingdom

**Keywords:** acetylcysteine, cell transplantation, hepatocytes, Liberase

## Abstract

**Background aims:**

Successful hepatocyte isolation is critical for continued development of cellular transplantation. However, most tissue available for research is from diseased liver, and the results of hepatocyte isolation from such tissue are inferior compared with normal tissue. Liberase and *N*-acetylcysteine (NAC) have been shown separately to improve viability of isolated hepatocytes. This study aims to determine the effect of Liberase and NAC in combination on human hepatocyte isolation from normal and diseased liver tissues.

**Methods:**

Hepatocytes were isolated from 30 liver specimens through the use of a standard collagenase digestion technique (original protocol) and another 30 with the addition of NAC and standard collagenase substituted by Liberase (new protocol). Viability and success, defined as maintenance of cell adhesion and morphology for 48 hours, were assessed. Metabolic function was assessed by means of albumin and urea synthesis.

**Results:**

Baseline factors were similar for both groups. The delay to tissue processing was slightly shorter in the new protocol group (median, 2 versus 4 hours; *P* = 0.007). The success rate improved from 12 of 30 (40.0%) to 21 of 30 (70.0%) with the use of the new protocol (*P* = 0.037), and median viable cell yield increased from 7.3 × 10^4^ to 28.3 × 10^4^ cells/g tissue (*P* = 0.003). After adjusting for delay, success rate (*P* = 0.014) and viable cell yield/g tissue (*P* = 0.001) remained significantly improved. Albumin and urea synthesis were similar or superior in the new protocol group.

**Conclusions:**

NAC and Liberase improve the success of hepatocyte isolation, with a significantly higher yield of viable cells. The use of these agents may improve the availability of hepatocytes for transplantation and laboratory research.

## Introduction

Recent years have seen a growing interest in cell therapy for various types of liver disease and, while research continues into the use of stem cells and their derivatives (1–5), several hurdles remain. The potential for tumor formation, the possibility that stem cells will not differentiate correctly after transplant or that stem cell–derived cells may not function in an identical manner to primary adult cells are all of concern as well as various ethical and moral issues (6–9). The use of fully differentiated primary hepatocytes overcomes some of the problems associated with stem cell therapy. There is, therefore, continued interest in hepatocyte transplantation as an alternative to orthotopic liver transplantation, particularly for certain inherited metabolic disorders. Although early experience has been encouraging, any clinical benefit has tended to be short-lived. Thus, hepatocyte transplantation is the subject of on-going laboratory and clinical research (10,11), and a supply of high-quality primary hepatocytes is crucial to further develop this treatment (12,13). In addition, primary hepatocytes continue to be a valuable resource for many aspects of biochemical and pharmaceutical research (14,15) as the limitations of hepatocyte cell lines are increasingly recognized.

Most studies of hepatocyte isolation describe the use of surplus donor tissue made available through liver transplant programs or normal tissue removed during resection of liver tumors (14,16–19). However, the majority of tissue available for research is from explanted diseased liver, which yields much fewer viable cells. We previously reported our experience of hepatocyte isolation from 100 normal and diseased livers, with a success rate of 51% and median viability of 40% (20). While demonstrating the potential of diseased and/or fatty liver tissue to provide functioning hepatocytes, these results are inferior to those obtained by centers that use donor and resected liver tissue alone. A means of improving the outcome of hepatocyte isolation from diseased liver would allow better use to be made of available liver tissue, increasing the pool of primary hepatocytes for research purposes.

The two-step collagenase digestion technique was first described in 1969 by Berry and Friend (21) for the isolation of rat hepatocytes and has since been adapted by several groups for the isolation of human hepatocytes. Two further adaptations have recently been separately described that may offer the potential to improve the outcome of hepatocyte isolation. Liberase is a relatively new enzyme initially developed to improve the outcome of pancreatic islet isolation (22–24). It comprises purified high–specific activity collagenases (clostridial collagenase I and collagenase II) blended with high–specific activity neutral protease (dispase or thermolysin) in an optimal ratio. Liberase blends are xeno-free, which is important for downstream applications. Little has been published on the use of Liberase for human hepatocyte isolation, but it has been shown to improve the viability of isolated porcine hepatocytes compared with standard collagenase (25). *N*-acetylcysteine (NAC) is an antioxidant that acts through the replenishment of hepatic glutathione stores. It also has direct antioxidant properties (26) and appears to have hepatoprotective effects through a number of mechanisms (27). Animal data suggest that NAC may have a protective effect against liver ischemia/reperfusion injury (28–30), and the use of NAC has recently been shown to improve the viability of human hepatocytes isolated from steatotic liver (31).

We report our experience of combining both Liberase and NAC in an attempt to improve the outcome of human hepatocyte isolation from normal and diseased liver tissue, comparing our results with those obtained with our previous protocol with the use of standard collagenase without NAC.

## Methods

### Ethics approval

All human liver tissue was obtained from Queen Elizabeth Hospital Birmingham or Birmingham Children's Hospital, with full approval of the Local Research Ethics Committee (reference No 06/Q702/61). Liver specimens were obtained from liver resections, whole explanted livers or donor liver, which was either surplus to surgical requirements or unsuitable for transplantation. Written informed consent was obtained from all patients or their families.

### Human hepatocyte isolation

Human hepatocyte isolation was performed as tissue became available over the 3-year period of January 2009 to 31 December 2011. Explanted livers were used from patients undergoing transplantation for any disease with the exception of those in whom we have previously found that high levels of hepatic necrosis preclude successful hepatocyte isolation. These include fulminant liver failure including drug toxicity and paracetamol overdose or those undergoing re-graft for acute rejection, primary non-function or hepatic artery thrombosis. We also excluded liver tissue from patients with viral hepatitis or human immunodeficiency virus infection because of the risks of infection associated with handling and culturing potentially infected tissue in the laboratory.

After explantation or resection, liver specimens were immediately placed on ice in a sealed sterile bag. Donor liver was retrieved and transported on ice in cold University of Wisconsin solution within a sterile bag according to standard protocols by the organ retrieval team. All donor liver tissue was assessed by the clinical team at Queen Elizabeth Hospital, Birmingham, and only made available for hepatocyte isolation if deemed unsuitable for liver transplantation. On arrival in the laboratory, liver tissue was processed immediately by a trained pathologist and hepatocyte isolation was carried out immediately thereafter. Where explanted or donor livers were used, wedges were obtained from either segments II/III, segments V/VI or segments VII/VIII. In the case of resected specimens, only those from patients undergoing right hemihepatectomy were deemed suitable by our pathologist to obtain sufficient tissue for isolation while not compromising the subsequent histopathological examination of the tumor and resection margins. Therefore, wedges from resection specimens were obtained from segments V/VI or VII/VIII. In all cases, tissue was only used for cell isolation if there was no indication of tumor in that part of the liver after inspection by the pathologist in conjunction with available preoperative imaging. All resection specimens were for colorectal metastasis, and all patients had received preoperative chemotherapy.

**Figure 1:**
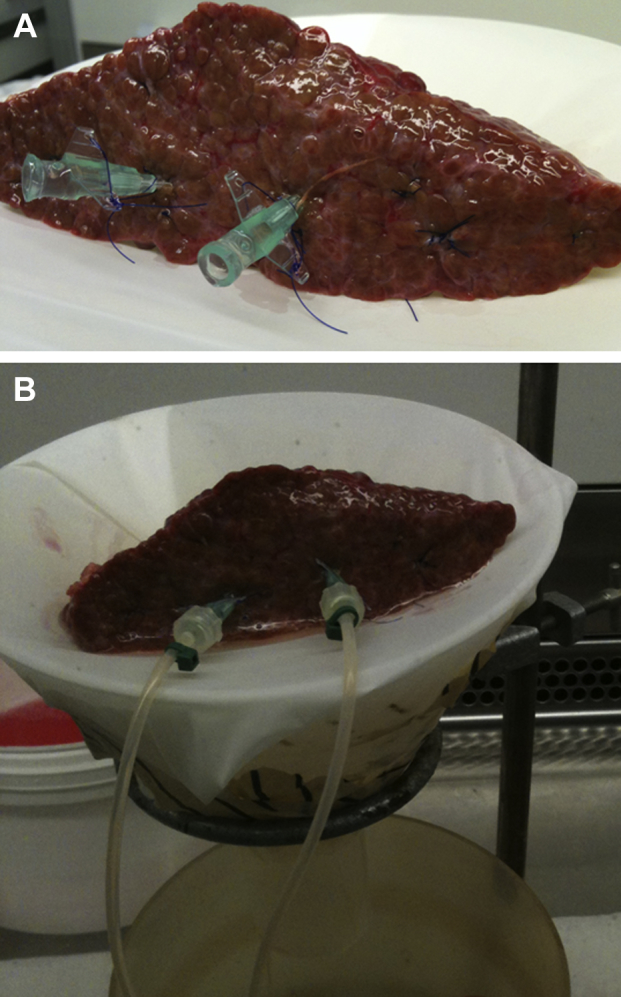
(A) Liver wedge prepared for perfusion with two cannulae inserted and the remaining vessels ligated with sutures and (B) connected to the perfusion circuit. The liver wedge was placed on a mesh-covered funnel and the perfusate was allowed to drain into a vessel placed beneath from where it was removed (wash and EGTA buffers) or recirculated (enzyme buffer).

Hepatocytes were isolated from 30 consecutive liver specimens as previously described (original protocol) (20). Briefly, once the liver wedge was cut, it was flushed with cold Dulbecco's modified Eagle's medium (DMEM) (Invitrogen Life Sciences, Paisley, United Kingdom) to remove any remaining blood and to identify vessels for cannulation. In each case, two vessels were identified such that maximum perfusion of the entire wedge was obtained. The vessels were cannulated with the use of two 18- or 20-gauge cannulas (Becton-Dickinson, Oxford, United Kingdom) and secured by means of a 4–0 Prolene (Covidien, Hampshire, United Kingdom) purse-string suture placed before insertion of the cannula. All remaining vessels on the cut surface of the liver were then closed with more sutures (Figure 1). Perfusion commenced with 500 mL of wash buffer at a flow rate of 75 mL/min in a non-recirculating fashion. This began the warming of the liver to the optimal temperature for enzymatic dissociation and ensured that any remaining blood was washed out. After this, the liver was perfused with 500 mL of 0.5 mmol/L ethylene glycol tetra-acetic acid (EGTA, Sigma-Aldrich, Dorset, United Kingdom) chelating buffer to remove divalent cations, thus disrupting cell-cell and cell-extracellular matrix adhesions. The liver was then perfused with another 500 mL of wash buffer to remove any remaining EGTA, which would otherwise inhibit the action of the enzymes that require the presence of calcium and magnesium. Finally, the liver was perfused with 300 mL of recirculating warmed buffer containing 0.5% wt/vol collagenase A (Roche, Hertford, United Kingdom), 0.25% wt/vol protease type XIV, 0.125% wt/vol hyaluronidase and 0.05% wt/vol deoxyribonuclease (all from Sigma-Aldrich) until adequate digestion was achieved. During this time, the appearance and texture of the liver was monitored closely. During enzymatic digestion, the liver tissue is seen to become paler in color and softer in texture as the parenchymal cells are dissociated. Perfusion was stopped once a digit could be inserted into the liver with minimal force. Once perfusion was complete, the cannulas were removed and the liver was placed in cold (4°C) DMEM supplemented with 10% vol/vol heat-inactivated fetal calf serum (Invitrogen Life Sciences) and 1% vol/vol penicillin/streptomycin/glutamine solution (10,000 units/mL penicillin, 10 mg/mL streptomycin and 200 mmol/L L-glutamine, Invitrogen Life Sciences) in a sterile glass dish. The liver was manually dissociated and the resulting suspension passed through a 250-μm sterile nylon mesh followed by 63-μm sterile nylon mesh (John Staniar & Co, Manchester, United Kingdom). The cell suspension was then washed three times and the hepatocytes were pelleted by means of low-speed centrifugation (50*g* for 5 minutes at 4°C) in supplemented DMEM. Cell viability was determined by means of trypan blue dye exclusion. If the viability was low but the total cell yield was sufficiently high, a Percoll (GE Healthcare, Buckinghamshire, United Kingdom) density gradient centrifugation step was performed to improve the yield of viable cells. Hepatocytes were then plated in Williams E media (Sigma-Aldrich) supplemented with 10% vol/vol fetal calf serum and 1% vol/vol penicillin/streptomycin/glutamine solution. Cells were seeded on type I rat-tail collagen-coated 24 well plates (5 × 10^5^ cells/well) or flasks and allowed to adhere for 3 hours. Seeding density was selected to ensure a confluent monolayer. After this period, the media was changed to a serum-free hepatocyte culture medium that was based on the long-term medium developed by Pichard *et al.* (32) and cells were maintained at 37°C in a humidified 5% CO_2_ incubator.

Another 30 consecutive liver specimens were perfused in an identical manner with the exception of two modifications. First, 5 mmol/L NAC (Sigma-Aldrich) was added to the EGTA-containing buffer. Second, standard collagenase was replaced by Liberase (Roche). Specifically, the Liberase TM blend was selected because this has been previously tested for rodent and porcine hepatocyte isolation according to information provided by the manufacturer. After initial attempts with the use of the recommended concentration for porcine hepatocyte isolation (25 μg/mL), a concentration of 33.3 μg/mL (10 mg in 300 mL of enzymatic buffer) was found to be optimal, and this concentration was used for all isolations with the use of the new protocol in this study. Liberase also contains a neutral protease, thermolysin, and therefore the neutral protease included in the original protocol was not included in the new protocol. All other aspects of the isolation process were unchanged from the original protocol.

All isolations were performed by a single operator (DCB) to exclude the possibility of inter-operator variability. Success was defined as maintenance of cell adhesion and morphology in a confluent monolayer for 48 hours.

### Albumin and urea synthesis assays

Albumin concentration was analyzed in tissue culture supernatants from normal, primary biliary cirrhosis (PBC)/primary sclerosing cholangitis (PSC) and alcoholic liver disease (ALD) hepatocytes at days 1, 3, 5 and 7, by means of a sandwich enzyme-linked immunosorbent assay kit (Abnova, Taipei City, Taiwan). Similarly, urea synthesis was confirmed by means of a quantitative colorimetric urea determination method (QuantiChrom Urea Assay Kit, Bioassay Systems, Hayward, CA, USA).

### Statistical analysis

Continuous data were compared through the use of Mann-Whitney tests. Fisher's exact test was used for categorical data. Albumin and urea synthesis were compared by means of repeated-measures analysis of variance (ANOVA). Both of these variables were log_10_-transformed before analysis to improve their respective distributions. Repeated-measures ANOVAs were then used to compare albumin and urea synthesis by hepatocytes isolated from different tissue types with the new protocol. The day of the measurement was used as the within-subjects factor and tissue type as the between-subjects factor. Tukey's honest significant difference test was used to make *post hoc* pairwise comparisons between the tissue types, to identify where any significant differences lay. Repeated-measures ANOVAs were then used to compare the effect of the new and original protocols on urea and albumin values. The day of the measurement was entered as a within-subjects factor and protocol as a between-subjects factor. Separate analyses were performed for each of the tissue types; hence, the critical *P* value was Bonferroni-adjusted to account for multiple comparisons. All analyses were performed with the use of IBM SPSS 19 (IBM Corp, Armonk, NY, USA), with a value of *P* < 0.05 considered to be indicative of statistical significance.

## Results

### Liver tissue and baseline factors

The underlying disease of the tissue used for cell isolation is shown in Table I and included PBC or PSC and ALD. Non-diseased tissue was obtained from the uninvolved (ie, tumor-free) tissue removed during resection of colorectal metastases (resected tissue) or was obtained from surplus donor tissue. The latter was rejected for transplant because of prolonged ischemic times and/or extensive steatosis. A few specimens, referred to here as normal, were obtained from unused portions of split livers or reduced livers used for pediatric transplant.

A number of baseline factors related to the liver tissue used for isolation in the two groups are shown in Table II. These include patient age and sex, Model for End-Stage Liver Disease (MELD) and United Kingdom End-Stage Liver Disease (UKELD) scores, delay from the time of liver explant/resection (or, for donor/normal tissue, from the time that the liver arrived at Queen Elizabeth Hospital, Birmingham) until commencement of perfusion and the weight of the wedge used for isolation. There was no significant difference in either the original diseases or any of the variables between the two groups, with the exception of time delay, which was significantly longer in the original protocol group (median, 4 hours versus 2 hours; *P* = 0.007).

### Effect of protocol modification on perfusion time and Percoll use

Although the isolation procedure remained unchanged other than the modifications being studied, the duration of perfusion (enzymatic digestion) and use of Percoll density gradient centrifugation varied, depending on the nature of the particular liver specimen and the initial cell yield and viability as described above. We therefore investigated the effect of the new protocol on these factors, and the results are summarized in Table III. The new protocol necessitated a slightly longer perfusion time than the original protocol (median, 4.5 versus 3.0 minutes; *P* = 0.028). However, there was no difference in the frequency with which the Percoll step was required (*P* = 0.792). Furthermore, when Percoll was used, the initial cell yield before Percoll was similar with the new protocol compared to the original protocol (median, 256 × 10^6^ versus 275 × 10^6^; *P* = 0.728), as was viability (median, 19% versus 18%; *P* = 0.839).

### Effect of protocol modification on outcome of hepatocyte isolation

The next stage was to compare a number of key outcomes between the two protocols. For continuous variables, comparisons between the groups were performed with the use of Mann-Whitney tests, with Fisher's exact test used for binary outcomes. In addition to this, the analyses were repeated with the use of general linear models or binary logistic regression, as appropriate, to adjust the *P* values to account for the fact that the delay differed significantly between protocols. These results are summarized in Table IV. Initial viability and cell yield (before Percoll) and final viability and cell yield (after Percoll, when it was used) are reported.

No effect was found on the absolute initial or final cell yield. However, the new protocol resulted in significant increases in both initial (*P* = 0.007) and final (*P* = 0.043) viability, with the median values rising from 10% to 25% and 48% to 70%, respectively. In addition to this, the final viable cell yield/g tissue showed a significant increase (*P* = 0.003), with the median rising from 7.3 × 10^4^ in the original protocol to 28.3 × 10^4^ in the new protocol. Success rates also increased significantly (*P* = 0.037), from 12 of 30 (40.0%) under the original protocol to 21 of 30 (70.0%) with the new protocol. After adjustment to account for the difference in the average delay between the protocols, the effect of the protocol on final viability ceased to be significant (*P* = 0.063). However, all other variables found to be significant in univariate analysis remained so after adjustment for the delay.

The effect of protocol modification on hepatocyte isolation from liver tissue of different disease types is summarized in Table V. There was an improvement in outcome for all liver tissue types, but the greatest benefit was seen with ALD and resected liver tissue. For ALD liver, the median final viable cell yield increased to 9.83 × 10^4^ with the new protocol, from 0.26 × 10^4^ with the old protocol (*P* = 0.04). The success rate also significantly improved to 63% with the use of the new protocol, whereas no isolations from ALD tissue performed with the use of the old protocol resulted in maintenance of a confluent monolayer in culture for 48 hours (*P* = 0.03). For resected tissue, the median final viable cell yield increased to 61.88 × 10^4^ with the new protocol, from 9.48 × 10^4^ with the old protocol (*P* = 0.02). The success rate increased from 33% to 100%, although this did not reach statistical significance.

### Morphology and phenotype of isolated hepatocytes

**Figure 2:**
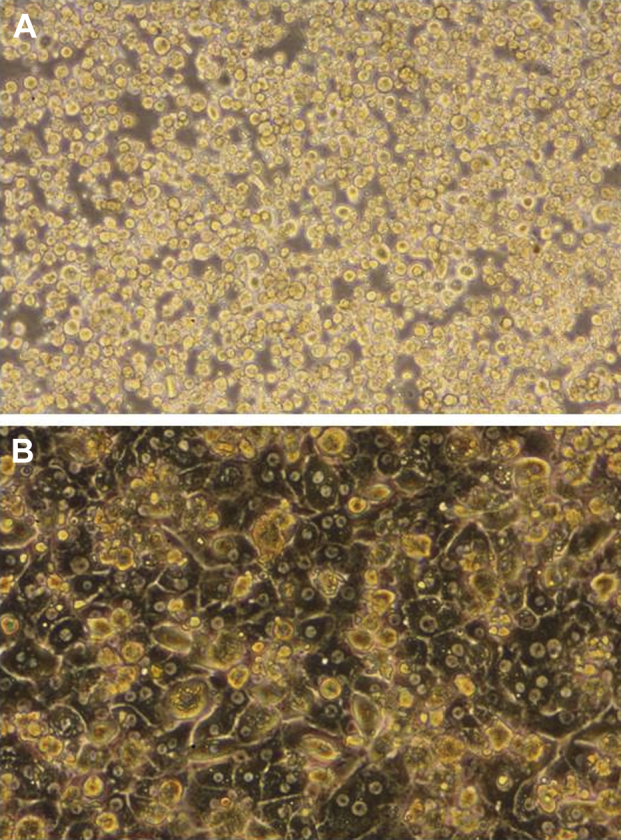
Morphology of human hepatocytes isolated from liver tissue. Primary human hepatocytes isolated from normal donor liver tissue with the use of the new protocol showing typical morphological changes: (A) 1 hour after plating, the cells are rounded and phase bright; (B) after 48 hours, a confluent monolayer has formed. The hepatocytes have flattened out and show refractive borders. The typical polygonal appearance is seen; many cells have two nuclei.

Human hepatocytes isolated with the use of the new protocol had an appearance similar to that previously reported by us and others (20,33). Figure 2 shows the morphology of hepatocytes isolated from normal liver tissue at different time points after isolation. On initial plating, cells appeared rounded and bright, but during the next 24 hours, cells gradually flattened out, forming a confluent monolayer and demonstrating a typical polygonal appearance, with many cells being binucleate. This morphology was maintained in culture for at least 1 week.

**Figure 3:**
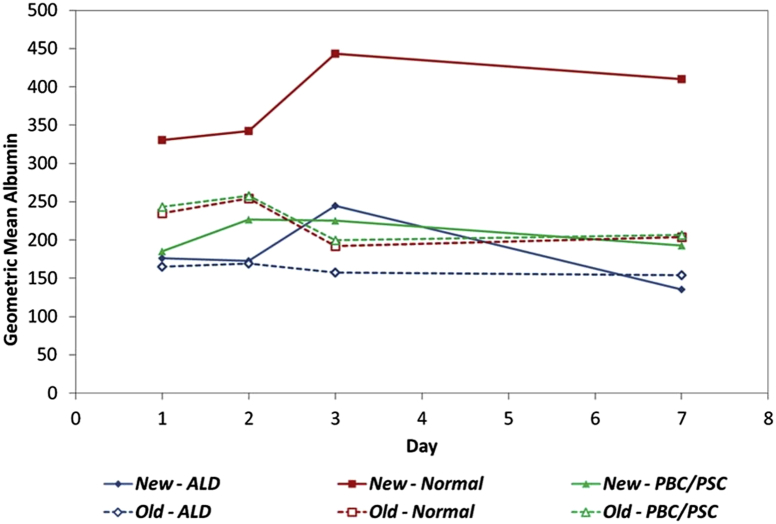
Albumin synthesis by primary human hepatocytes isolated from normal and diseased liver. Solid lines represent new protocol; broken lines represent original protocol.

Albumin synthesis by hepatocytes isolated from normal, PBC/PSC or ALD liver tissue with the use of the modified protocol was maintained for at least 1 week in culture (Figure 3). Underlying liver disease was found to have a significant effect on albumin synthesis (*P* < 0.001, Table VI), with synthesis by hepatocytes from normal liver significantly higher than both ALD and PBC/PSC (both *P* < 0.001). There was no significant difference between the albumin measurements of ALD and PBC/PSC hepatocytes (*P* = 0.187).

We next compared albumin synthesis by hepatocytes isolated by means of the new and original protocols. There was no significant difference in albumin values for ALD tissue (*P* = 0.053). However, for the other two tissue types, significant changes were detected. For hepatocytes isolated from normal tissue, the geometric mean albumin measurements increased from 220 under the old protocol to 379 with the new protocol (*P* < 0.001). For PBC/PSC, on the other hand, the new protocol caused a significant reduction in albumin, from 226 under the old protocol to 207 under the new one (*P* = 0.012).

**Figure 4:**
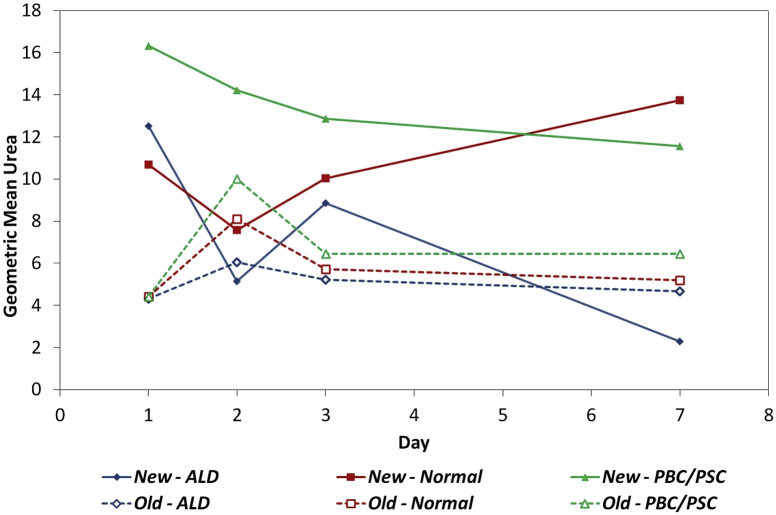
Urea synthesis by primary human hepatocytes isolated from normal and diseased liver. Solid lines represent new protocol; broken lines represent original protocol.

Urea synthesis by hepatocytes isolated from normal, PBC/PSC or ALD liver tissue with the use of the modified protocol was maintained for at least 1 week in culture (Figure 4). Tissue type was found to have a significant effect on urea measurements (*P* = 0.002, Table VI). The average ALD urea measurements were significantly lower than both normal (*P* = 0.006) and PBC/PSC (*P* = 0.002). There was no significant difference between the urea measurements from hepatocytes isolated from normal and PBC/PSC tissue (*P* = 0.778).

We next compared urea synthesis by hepatocytes isolated by means of the new and original protocols (Table VII). The results showed no significant difference in urea values between the two protocols for ALD tissue types (*P* = 0.159). However, significant increases were brought about by the new protocol for both normal and PBC/PSC tissue (*P* = 0.001 and 0.002, respectively).

## Discussion

At a time when the discrepancy between the need for organ transplant and the supply of donor organs is growing (34), the potential for treatments such as hepatocyte transplantation to delay or avoid the need for orthotopic liver transplantation cannot be ignored. Therefore, the demand for high-quality human hepatocytes for cell transplantation as well as pharmacological and toxicological studies continues to rise, yet the availability of liver tissue for research remains limited. Our previous study of hepatocyte isolation from more than 100 liver specimens, of which 54% were from cirrhotic, end-stage liver diseases, showed for the first time that viable functioning hepatocytes may be routinely isolated from diseased liver (20). In that series, our overall success rate was 51%, with a median viability of 40%. While we successfully isolated cells from all types of liver disease, we showed that ALD livers produced the poorest results and that cells isolated from ALD livers had inferior metabolic function in terms of albumin and urea synthesis. Furthermore, we showed that time delay between hepatectomy/explant and the commencement of perfusion influenced the likelihood of success.

Although the results from our previous series were encouraging, the viability achieved fell short of that achieved by groups that used more favorable tissue, and, in nearly half the cases, the outcome was not successful. We therefore set about improving the viability and cell yield, focusing on the use of Liberase and NAC because other studies in the literature suggested that they may be of benefit. Donini *et al.* (25) investigated the outcome of hepatocyte isolation from 14 porcine livers randomly assigned to standard collagenase or Liberase. Mean cell viability in that study was extremely high even without the use of Liberase (90%), most probably because livers were retrieved from normal healthy pigs and perfused *in situ* with a cold preservation solution before immediate progression to hepatocyte isolation. Liberase significantly increased mean viability to 95%, although there was no effect on cell yield. Sagias *et al.* (31) isolated hepatocytes from 10 severely steatotic (>60%) livers, with two specimens from each liver randomly assigned to standard collagenase digestion or the same digestion technique with the addition of NAC. Use of NAC significantly increased mean viability from 66% to 81% as well as the mean viable cell yield from 1.10 × 10^6^ cells/g tissue to 2.59 × 10^6^ cells/g tissue.

We decided to combine the use of both Liberase and NAC at the same time in an attempt to optimize the impact on hepatocyte isolation. Because liver tissue is in high demand within our laboratory for the isolation of various non-parenchymal cells, we were unable to randomize tissue from the same liver to each of the two protocols. However, our approach has enabled us to produce the largest series to date investigating the use of Liberase and NAC and, to the best of our knowledge, the only series to use both in combination. Furthermore, we know of no other study describing the use of these reagents to isolate human hepatocytes from a wide range of diseased and cirrhotic livers. Our data confirm that the use of Liberase and NAC in combination significantly improves the outcome of human hepatocyte isolation from normal and diseased liver in terms of both viable cell yield and overall success rate.

The time delay until the start of the isolation procedure was significantly shorter in the new protocol group, and this is likely to reflect improvements that have been made to the arrangements for collecting liver specimens from the hospital and their subsequent processing. These improvements occurred during the period when we performed the first few isolations included in the study; the median delay to processing for the second half of the old protocol group (specimens 16–30) was 2 hours, the same as for the new protocol group. Despite this, there was no trend to improved outcomes during the first 30 patients (old protocol), suggesting that an improvement in time delay alone was not responsible for the improved outcome with the new protocol. This is further demonstrated by the fact that even after adjusting for the difference in time delay between the two groups, the outcomes were still significantly improved.

The enzyme perfusion time required for digestion with the new protocol was slightly longer than with the old protocol. Perfusion time is not pre-determined; rather, perfusion continues until adequate tissue digestion is achieved. As such, it is a function of both the nature of the liver tissue being digested and the specific enzyme cocktail being used. Although there was no significant difference in the range of liver disease types used between the two groups overall, there was possibly more cirrhotic liver in the new protocol group (slightly more biliary cirrhosis and ALD liver). We also suspect that Liberase has a gentler action than did standard crude collagenase preparations and may require a longer time to act. Indeed, other authors have reported longer digestion times for other tissues such as pancreas (35) and ovary (36) when using Liberase compared with standard collagenase.

The decision to use Percoll is somewhat subjective and in our experience always results in a loss of cells above what would be expected, given the initial cell viability and yield. Therefore, we only use this step when there are a large number of cells with very low viability. The fact that Percoll use was similar between the two groups suggests that we were consistent in the application of criteria for its use and that differential use of Percoll does not account for the improvement seen with the new protocol.

The fact that we have shown an improvement in overall success rate, which we defined as the maintenance of cell adhesion and morphology for 48 hours, as well as viable cell yield is important. Many experimental protocols require cells to be plated down and maintained in culture for a period of time; therefore an increased yield of viable cells that fail to attach in culture would be of limited benefit. Furthermore, hepatocytes are known to undergo anoikis or detachment-related cell death (37,38); therefore, failure to attach will quickly render isolated hepatocytes useless. Even if used immediately for cell transplantation, it seems likely that such cells would be less able to survive and engraft (39). It has been shown that establishment of a confluent monolayer is critical for maintenance of the differentiated hepatocyte phenotype (40); therefore we used the presence or absence of a confluent monolayer, rather than attempting to quantify adhesion, to determine success. A 48-h culture period was a pragmatic choice that would be likely to provide sufficient time for downstream experiments or use of the hepatocytes for transplantation without the loss of phenotype associated with prolonged culture.

It is interesting that the new protocol did not result in an increase in total cell yield. The previous studies of NAC or Liberase in hepatocyte isolation also reported no increase in absolute cell yield (25) or only reported an increase in viable cell yield (31). It is therefore likely that the beneficial effects of both these agents are exerted mainly through an improvement in viability; indeed, it is difficult to see how the antioxidant and hepatoprotective actions of NAC might improve absolute cell yield. Obtaining a high cell yield depends on sufficient tissue digestion, and we continue enzyme perfusion until the liver can be easily manually dissociated rather than setting a fixed perfusion time for all types of liver. We believe that this approach already maximizes the absolute cell yield obtained but that the continued exposure of the hepatocytes to enzymes during this time is detrimental. Therefore, it is likely that an improved enzyme preparation that is less damaging to individual cells will improve final viability and viable cell yield but not the overall number of cells obtained.

In this study, we confirmed our previous findings that hepatocytes isolated from ALD liver generally have poorer metabolic function compared with those isolated from normal or PBC/PSC liver. The metabolic function of hepatocytes isolated with the use of the new protocol was similar to that of hepatocytes isolated with the use of the original protocol for ALD hepatocytes and generally improved for normal and PBC/PSC hepatocytes. Although PBC/PSC hepatocytes showed a lower average albumin synthesis, this result is perhaps explained by the higher albumin values for PBC/PSC hepatocytes isolated by means of the original protocol during the first 48 hours of culture; by day 7, the values for both groups were similar. There are many other aspects of hepatocyte function that we have not investigated, and we have not attempted to show maintenance of the complete hepatocyte phenotype in culture. However, these results are encouraging because they suggest that the improved overall success rate and viable cell yield do not occur at the expense of metabolic function. It is also of note that the greatest improvement in metabolic function was seen in hepatocytes isolated from normal liver tissue; these cells are most likely to be used for clinical purposes and are preferred for laboratory investigation.

In conclusion, we report that the combined use of NAC and Liberase for the isolation of human hepatocytes from normal and diseased liver results in a higher success rate and viable cell yield. Furthermore, metabolic function is maintained for up to 1 week in culture and is improved compared with hepatocytes isolated without NAC and Liberase. The routine use of NAC and Liberase should greatly increase the availability of primary human hepatocytes both for research and clinical applications.

## Figures and Tables

**Table I:** Liver tissue from a similar variety of diseases was used for hepatocyte isolation in each of the study groups with no significant difference in the range of diseases between each group.

Disease	Original protocol	New protocol
Biliary cirrhosis (primary biliary cirrhosis or primary sclerosing cholangitis)	5	7
Alcoholic liver disease	6	8
Resected tissue	6	4
Normal	2	1
Donor tissue	8	5
Other	3	5

*P* = 0.77.

**Table II:** Baseline factors.

Factor	Original protocol	New protocol	*P* value
Age, y	57.5 (38.0–65.8)	56.5 (45.0–62.0)	0.997
Sex, male^a^	18 (60.0%)	17 (56.7%)	0.600
UKELD	56.5 (50.3–58.8)	54.5 (49.3–59.8)	0.494
MELD	18.0 (12.8–21.3)	14.5 (9.3–19.0)	0.254
Delay, h	4.0 (2.0–19.0)	2.0 (1.0–3.0)	0.007^b^
Weight of wedge, g	83.5 (58.3–125.1)	88.7 (67.5–107.3)	0.502

Data displayed as median (quartiles) and *P* values from Mann-Whitney tests unless stated otherwise. Analyses were also repeated parametrically, where applicable, and returned comparable results.

a Data displayed as n (%) and *P* values from Fisher's exact tests.

b Significant at *P* < 0.05.

**Table III:** Effect of isolation protocol on perfusion time and Percoll use.

Factor	Original protocol	New protocol	*P* value^a^
Perfusion time, min	3.0 (2.5–6.1)	4.5 (4.0–6.0)	0.028^b^
Percoll used^a^	13 (43.3%)	11 (36.7%)	0.792
Cell yield before Percoll, ×10^6^	275 (77.5–474.8)	256 (115.0–317.5)	0.728
Viability before Percoll	18% (11.3–32.5%)	19% (13.0–26.0%)	0.839

Data for perfusion time displayed as median (quartiles) and *P* values from Mann-Whitney tests unless stated otherwise. Analyses were also repeated parametrically and returned comparable results.

a Data displayed as n (%) and *P* values from Fisher's exact tests.

b Significant at *P* < 0.05.

**Table IV:** Effect of protocol modification on initial (before Percoll) and final (after Percoll) cell yield and viability and overall success.

Factor	Original protocol	New protocol	Unadjusted *P* value^a^	Adjusted *P* value^b^
Initial yield/g tissue, ×10^6^	105.1 (28.2–400.8)	125.0 (41.5–277.6)	0.775	0.872
Initial viability	10.0% (0.0–31.3%)	25.0% (14.5–56.3%)	0.007^c^	0.015^c^
Final cell yield, ×10^6^	29.0 (12.4–104)	42.5 (15.9–119.4)	0.478	0.236
Final yield cells/g tissue, ×10^4^	27.5 (18.9–134.1)	48.5 (16.2–114.4)	0.654	0.289
Final viability	48.0% (0.0–80.0%)	70.0% (23.2–83.1%)	0.043^c^	0.063
Final viable cell yield/g tissue, ×10^4^	7.3 (0.0–20.5)	28.3 (8.2–70.2)	0.003^c^	0.001^c^
Success	12 (40.0%)	21 (70.0%)	0.037^c^	0.014^c^

Data displayed as median (quartiles) for continuous variables and n (%) for binary outcomes.

a *P* value from Mann-Whitney or Fisher's exact test for continuous and binary outcomes, respectively.

b *P* value from a general linear model or binary logistic regression, for continuous and binary outcomes, respectively, after adjustment for the effect of the time delay.

c Significant at *P* < 0.05.

**Table V:** Effect of isolation protocol on final viable cell yield and success rate of hepatocyte isolations from different types of liver tissue.

Disease type	Final viable cell yield/g tissue, ×10^4^		Success	
	Old protocol	New protocol	Old protocol	New protocol
ALD	0.26 (0.00–0.90)	9.83 (3.51–31.61)	0%	63%
Biliary cirrhosis	10.59 (5.48–20.34)	29.12 (17.23–85.78)	60%	71%
Resected	9.48 (0.00–20.39)	61.88 (52.26–97.54)	33%	100%
Normal/donor	11.52 (4.88–24.76)	16.57 (9.31–38.55)	60%	83%

Data displayed as median (quartiles) for continuous variables.

**Table VI:** Albumin and urea concentration in tissue culture supernatants of hepatocytes isolated different types of liver isolated with use of the new protocol.

	Tissue type	Geometric mean (95% confidence interval)	Tukey's honest significant difference test, *P* value vs		
			ALD	Normal	PBC/PSC
Albumin	ALD	176 (159–195)	–	<0.001^a^	0.187
	Normal	368 (326–415)	<0.001^a^	–	<0.001^a^
	PBC/PSC	199 (180–221)	0.187	<0.001^a^	–
Urea	ALD	5.6 (4.3–7.2)	–	0.006^a^	0.002^a^
	Normal	10.7 (8.3–13.8)	0.006^a^	–	0.778
	PBC/PSC	12.0 (9.3–15.4)	0.002^a^	0.778	–

a Significant at *P* < 0.05.

**Table VII:** Comparison of albumin and urea concentration in tissue culture supernatants of hepatocytes isolated with use of the new and original protocols.

	Tissue type	Geometric mean (95% confidence interval)		*P* value
		Original protocol	New protocol	
Albumin	ALD	161 (150–173)	178 (166–191)	0.053
	Normal	220 (208–233)	379 (355–404)	<0.001^a^
	PBC/PSC	226 (216–235)	207 (198–216)	0.012^a^
Urea	ALD	5.0 (4.1–6.1)	6.0 (5.0–7.3)	0.159
	Normal	5.7 (4.7–6.7)	10.3 (8.8–12.1)	0.001^a^
	PBC/PSC	6.5 (5.2–8.2)	13.6 (10.8–17.2)	0.002^a^

a Significant after Bonferroni correction for three comparisons (*P* < 0.0167).
